# How Growing up with a Sibling with Disabilities Shapes Career Choices: A Qualitative Study of Helping Professionals in Japan

**DOI:** 10.3390/bs16060924

**Published:** 2026-06-04

**Authors:** Yoshimi Yano, Kyoko Nagata, Koji Tanaka

**Affiliations:** 1 Faculty of Nursing Science, Osaka Seikei University, Osaka 533-0007, Japan; yano-y@osaka-seikei.ac.jp; 2 Faculty of Health Sciences, Institute of Medical, Pharmaceutical and Health Sciences, Kanazawa University, Kanazawa 920-0942, Japan; ktanaka@staff.kanazawa-u.ac.jp

**Keywords:** siblings, disability, career choice, qualitative study, helping profession, Japan

## Abstract

Having a sibling with a disability is known to significantly impact career choices. However, the experiences and emotional processes of such individuals who pursue a career in a helping profession are not fully understood. This study aimed to clarify how the experience of growing up with a sibling with a disability in Japan has influenced their decision to pursue a career in a helping profession, as well as their approach to life as a helping professional. Following a descriptive qualitative approach, semi-structured interviews were conducted with people with a sibling with a disability, and the results were analyzed qualitatively and inductively. The participants did not necessarily have a clear motivation to pursue a career in a helping profession; rather, they were influenced by their family experiences and living environments from childhood, as well as emotional conflicts. Supporting clients and their families enabled them to deepen their understanding of disabilities and welfare, as well as to resolve their own unresolved issues. The findings of this study highlight the need for support for siblings of people with disabilities. Providing siblings with opportunities to reflect on their experiences and find meaning in them is considered important for supporting self-understanding and well-being.

## 1. Introduction

It is estimated that roughly 1.3 billion people worldwide—about 16% of the global population—live with a disability (WHO, 2023). Many of these individuals live with their families and have siblings, suggesting that the number of siblings of people with disabilities is even larger. It has been noted that people with a sibling with a disability have a variety of unique experiences throughout their lives (Meltzer, 2021).

Previous research has reported that growing up with a sibling with a disability can have implications for the personal, social, romantic, and occupational aspects of life (Milevsky & Singer, 2022; C. E. Lee et al., 2021). These experiences may involve complex and conflicting emotions regarding the sibling’s disability (Leane, 2019), and studies have shown that the effects can extend to a wide range of areas, including physical and mental health, financial circumstances, career choices, future caregiving plans, and quality of life (Wright et al., 2024).

At the same time, the experience of growing up with a sibling with a disability is not entirely negative. Previous research has reported that siblings often demonstrate high levels of resilience and belong to organizations that provide social support (C. E. Lee & Shikarpurya, 2025), and many maintain good relationships with their siblings and intend to remain involved in their lives even after reaching adulthood (Skotko et al., 2011). Furthermore, it has been noted that siblings may assume multiple roles, including friend (playmate), teacher, supporter, and caregiver (Lynam & Smith, 2022), and they may eventually take on a parental role, feeling a sense of responsibility to provide support and care.

From the perspective of family systems theory, family members influence one another through ongoing interactions within the family system, and raising a child with a disability affects the entire family (Noonan et al., 2018). Siblings are typically the family members who maintain the longest-lasting relationships with the person with a disability, and it is believed that their development is influenced by this relationship (Jones et al., 2019). Furthermore, the impact of these experiences changes across developmental stages. Although emotional problems associated with experiences of discrimination are commonly observed during childhood, previous research has indicated that situational constraints related to relationships with people with disabilities—such as future marriage and career—emerge as challenges from adolescence onward (Park et al., 2021).

As in many Asian countries, caregiving has traditionally been viewed in Japan as a responsibility that falls to the family. This responsibility is expected to be borne primarily by women (K. S. Lee, 2010). Although these norms have been shifting in recent years due to changes in family structures and social welfare systems, it has been noted that the responsibilities and role expectations placed on family members continue to influence family experiences (Sato et al., 2015). This cultural background may influence the sense of responsibility and perception of support roles among individuals with siblings with disabilities, as well as their future career choices.

Thus, it has been suggested that experiences as a sibling may influence important life decisions and are related to career paths, occupational choices, and future life planning (Milevsky & Singer, 2022). According to Alon (2026), siblings of individuals with autism spectrum disorder are significantly influenced by the key challenges of adulthood, including leaving the family home, finding a partner, early parenthood, career choices, concerns about the future, and identity formation.

Career choice is one of the most important decisions in a person’s life and is shaped by various factors, including personality, educational experiences, perceptions of gender roles, and interactions with parents and peers (Eccles, 2011).

Previous research has reported that many people with a sibling with a disability recognize that this experience has influenced their career choices (Milevsky & Singer, 2022; Nguyen et al., 2024). The experience of being a sibling can sometimes serve as motivation to pursue careers supporting children and young people with disabilities in fields such as healthcare, social welfare, and research (Nguyen et al., 2021). It has also been reported that around half of the people with a sibling with an intellectual disability are employed in caregiving or support-related professions (Beffel et al., 2023). Recent research has explored the experiences and motivations underlying the career choices of people who have siblings with disabilities (Alon, 2026). However, many studies have tended to treat career choice as an outcome, and the specific experiences, thought processes, and emotional journeys that led these individuals to pursue a career in a helping profession have not been sufficiently elucidated. Furthermore, little attention has been paid to how this decision affects their professional ethics, their approach to providing support, and even their own way of life.

“Helping profession” is defined as an occupation that involves professional interactions between experts and clients and promotes personal growth or addresses physical, psychological, intellectual, and emotional conditions through fields such as medicine, nursing, psychotherapy, psychological counseling, social work, education, coaching, and other related fields (Noor et al., 2025). It has been widely reported that individuals with siblings with disabilities often assume caregiving roles from an early age (Hajnalka & Ildikó, 2025). Therefore, this study focused on the medical, health, and welfare sectors—where supporting others is a primary job responsibility—to examine the relationship between these experiences and professional practice.

## 2. Methodology

### 2.1. Aim

This study aims to clarify how the experience of having a sibling with a disability influences the decision to pursue a career in a helping profession, as well as the professional identity, values, and perspectives on life of those working in the helping professions.

### 2.2. Design

This study follows the descriptive qualitative approach proposed by Sandelowski (2000). This method ensures that researchers remain faithful to the inherent meaning of the data and the nature of the participants’ discourse, without applying overly theoretical interpretations, thereby guaranteeing that the findings can serve as a guide for future policy and practice (Sandelowski, 2000). Because the purpose of the present study was to explore the experiences of people with a sibling with a disability through interviews and to provide initial insights that could serve as guidelines for future support and education, a qualitative descriptive approach was deemed most appropriate.

### 2.3. Study Participants and Settings

Recruitment involved purposive sampling. Participant recruitment was conducted using intentional sampling. The researchers explained the purpose and methods of the study to the directors of three institutions with whom they had prior professional connections and asked them to introduce potential participants. They also asked the institutions to display posters and distribute flyers with information about the study. Eight individuals who spoke with the directors or saw the posters contacted the researchers (five by email and three by phone). The researchers explained in detail the purpose and methods of the study again and confirmed their willingness to participate. During the interviews, two of the eight participants introduced someone they knew, and both of those individuals also joined the study. All participants were fully informed that participation in the study was voluntary and that there would be no disadvantage if they declined. No one withdrew from the study at any point.

### 2.4. Inclusion Criteria

The inclusion criteria for participation were as follows: (1) having a sibling with a physical, mental, or intellectual disability; (2) having experience living with the sibling with a disability; (3) being employed in a field related to healthcare, public health, or social welfare; and (4) age 18 years or older.

### 2.5. Data Collection

Semi-structured interviews were conducted in person from September 2022 to June 2023. All interviews were conducted in Japanese by the first author, a doctoral student trained in qualitative interview techniques who had no prior relationship with the participants. With the participants’ permission, the interviews were audio-recorded and lasted between 47 and 77 min. To ensure confidentiality, the interviews were held in a private room provided by the researchers. When participants found it difficult to travel to the designated location, a secure remote conferencing system was used.

The researcher first explained the study’s purpose, rationale, methodology, and ethical considerations. After obtaining informed consent once again, demographic information was collected from participants prior to the interview, including age, gender, family structure, the ages of the siblings, and current occupation. Participants were then encouraged to speak freely about the topics listed in Table 1.

The interview guide was developed through consultation among the researchers. During the interviews, we ensured that researcher biases did not influence the data collection by requiring researchers to suspend their own opinions and to set aside assumptions, past knowledge, and understandings of phenomena (Ashworth, 1996). Audio recordings were made using an IC recorder.

### 2.6. Data Analysis

Following the methods defined by Sandelowski (2000) and Holloway and Wheeler (2002), we created verbatim transcripts from the audio data obtained through interviews and read them repeatedly. We described the data in accordance with each participant’s life course in order to gain a comprehensive understanding of the data. We identified data descriptions in the verbatim transcripts related to emotions and perceptions that led to career choices and the decision to enter the helping professions, and coded them using short phrases. Coding was conducted independently by the first and second authors. Next, we integrated the data from all participants, identified similarities and differences in the meanings of the codes, and inductively organized and grouped the codes in order to extract subthemes. Furthermore, we compared the similarities and differences among the subthemes in order to generate potential themes. The first and second authors jointly extracted subthemes and themes, collaboratively reviewed the coded excerpts and new themes, and reached consensus on the themes through repeated data analysis and discussion. Subsequently, when the three authors coded the data from the 10th participant and compared it with the subthemes, they confirmed that the extracted meanings were repetitions of previously identified content. At that point, data saturation was considered to have occurred (Hennink & Kaiser, 2022).

Finally, we obtained validation from 3 participants to verify that the findings accurately reflected their experiences. No software was used for the data analysis in this study.

### 2.7. Ethical Consideration

This study was conducted with the approval of the Institutional Review Board at the author’s affiliated institution (Approval No.: 711161-1; Approval Date: 25 July 2024). It complies with the ethical guidelines for medical research involving human subjects as stipulated in the Declaration of Helsinki and its subsequent amendments. Participants received written and verbal explanations regarding the background, objectives, significance, and methods of the study, and were explicitly assured of their voluntary participation, the right to withdraw or revoke consent without disadvantage, and the protection of their privacy. Written informed consent was obtained from all participants. Personally identifiable information was anonymized or coded. During the interviews, care was taken to avoid causing participants psychological stress, and the participants were assured that they were not required to answer questions they did not wish to answer and that they could stop the interview at any time. The first author observed the participants for any signs of hesitation, reluctance, or distress during the interviews. If a participant experienced distress during the interviews and required psychological support, the first author was prepared to consult with a psychiatric nursing specialist or physician at their university. All materials related to the participants were strictly controlled and stored in a locked facility.

### 2.8. Rigor and Reflexivity

In this study, we endeavored to ensure credibility, transferability, dependability, and confirmability (Lincoln & Guba, 1985) as follows. (1) The first author is a doctoral student in nursing who, after attaining 7 years of clinical experience, is presently employed as a nursing faculty member at a university. This background was beneficial in gaining insights from the participants’ narratives. (2) We built trust with the participants by comprehensively explaining the research objectives and providing a friendly, relaxed environment. (3) The researchers discussed the results of the data analysis, fostering new perspectives. (4) The analytical results were verified by obtaining confirmation from participants. (5) By thoroughly describing the phenomena, the researchers endeavored to ensure generalizability, enabling readers to judge the extent to which the results could be applied to other time periods, environments, situations, and individuals. (6) Throughout all stages of the research, attention was paid to the impact on the researchers’ knowledge construction. Verifiability was ensured by setting aside preconceptions and striving to understand the participants’ lifeworld.

## 3. Results

### 3.1. Characteristics of Participants

The characteristics of the 10 participants are presented in Table 2. Nine participants (A–I) were female, and one (J) was male. An example coding tree is shown in Table 3.

### 3.2. Theme 1: By Chance

“By chance” refers to a situation in which the participants feel they have entered the human services field not out of strong personal desire or due to recommendations from others, but rather because, in a sense, they have become human services professionals by chance. At the time they entered this field, they did not have clear answers as to why they wanted to become a helping professional or what they wanted to achieve in that role. Participants described their decision to pursue a career in a helping profession as a natural progression, but ultimately, they were guided down this path by the awareness and conflicts cultivated through their lives with their siblings and other family members. Throughout their life experiences, from childhood to choice of career, they implicitly understood their position and seemed to be bound by unconscious constraints. This theme comprised two subthemes: (1) Not having strong motivation, and (2) Not forced.

#### 3.2.1. Subtheme 1: Not Having Strong Motivation

‘Not having a strong motivation’ means that, although the participants did not have a firm resolve to pursue a career as a helping professional, they ultimately chose that path.

Participant A, who lived with her older brother—who has both intellectual and physical disabilities—until he was 5 years old, recalled that as a child she used to imagine that she would become a nurse and her brother a doctor, and that she would cure his disability. Although she did not hold onto that dream indefinitely, she said that when she began thinking realistically about her career options in high school, she realized that a career in social services was an option.

“I think having an older brother with disabilities probably made social services feel very familiar to me. At first, rather than wanting to cure my brother, I think—perhaps because I was just a child—I might have felt that it would make my parents happy.”

(Participant A)

Regardless of the type of disability their siblings had, many participants reported that they “had never thought about the future” or “didn’t have any career aspirations.” However, when the time came to choose a higher education or career path, they explained that they ultimately chose careers in human services because they were close with their siblings with disabilities, and cited reasons such as “there was a school for social welfare nearby,” “my sibling lived nearby,” or “I had watched staff caring for people with disabilities since I was little”.

“Up until then, I think I was just living life without giving it much thought—just having fun and not really thinking about what I wanted to be. I was just taking things as they came. Since there was a teachers’ college in City A, I decided to aim for it, even though I wasn’t sure if I’d get in. And luckily, I was accepted into the early childhood education department at City A’s teachers’ college.”

(Participant B)

#### 3.2.2. Subtheme 2: Not Forced

‘Not forced’ means that they did not choose a career in a helping profession because their family encouraged them to, but rather that they ended up choosing this path after observing the lives of their parents and siblings up close.

The participants, having seen firsthand how their families cared for siblings with disabilities, developed a desire to help their parents. Their relationship with their parents influenced their perceptions. They behaved in ways that would not cause their parents trouble or worry, playing the role of the “good child” in their parents’ eyes. Although the participants cited general reasons such as “there were social workers nearby” or “there was a university nearby,” some of them also harbored a latent desire to satisfy their parents’ expectations.

“I didn’t really have anything I wanted to do, and I had no idea what I was capable of. But when it came time to choose a high school, I talked to my parents and told them, this school has a social welfare program, so I’m going there,’ and that’s how I decided on social welfare. My family didn’t tell me to think about my younger brother or the family’s future—they just told me to do what I liked.”

(Participant D)

Parents took care not to burden the participants with the responsibility of looking after their siblings. Because they felt they were “making the participants put up with” their siblings, they told them, “You’re free to do as you please,” and reportedly never said or did anything that would force them down a particular path in life. However, despite this tolerant and supportive attitude, the participants still sensed their parents’ expectations.

“I think my parents felt guilty toward my younger sister. Even when she would cry constantly, they couldn’t bring themselves to scold her. So I made sure not to do anything that would get me in trouble. When I first told them I wanted to go into social work, my mother said, ‘Just do what you love.’ Maybe she thought I was carrying a heavy burden, but she told me to just do what I wanted to do.”

(Patient H)

### 3.3. Theme 2: Solving Personal Challenges

‘Solving personal challenges’ refers to a situation in which living with a sibling with a disability meant being constantly subjected to the pressure to be a “good child,” resulting in unresolved desires and frustrations with society. Through interacting with service users as helping professionals, the participants found opportunities to address these unresolved issues, gaining job satisfaction in the process. Through their work, they strove to deepen their knowledge of disabilities and social welfare. This was not merely to support their own siblings but to improve social welfare services for service users and their families. This reaffirmed their choice to become a helping professional. This theme comprised three subthemes: (1) the impact of living with a sibling with a disability from an early age, (2) pursuing an understanding of disabilities and social welfare, and 3) discovering one’s own identity.

#### 3.3.1. Subtheme 1: Conflicts from an Early Age

‘Conflicts from an early age’ refers to the situation in which participants struggled between their desire to accept their sibling with a disability while living together with them and their difficulty in doing so.

During their early childhood, the participants’ experiences with their siblings and family members constituted their entire world, and these experiences shaped their growth and perception. Upon entering elementary school, the emergence of social connections created a gap in the participants’ perceptions. The participants became acutely aware of the discrepancy between their own perception and reality, stemming from their relationships with classmates and siblings, and the fact that their siblings belonged to different schools or classes than them.

Participant I began to feel that she should refrain from talking about her sister because a friend who came over to her house was shocked upon seeing her sister, who has cerebral palsy, and subsequently changed her attitude toward her. These feelings of anger and embarrassment toward those around her persisted even after she entered the workforce.

“The day after my friend saw my sister, when I went to school, word had already spread. From the morning, some kids were saying things like, ‘Your sister has a disability,’ and the rumor that ‘it’s better not to talk to her’ had spread. I felt the atmosphere in the class was heavy all day. After that, I stopped inviting friends over to my house”.

Meanwhile, Participant J felt anger and shame when people stared with curiosity at his sister with an intellectual disability, choosing to pretend she didn’t exist.

“I can say this now, but to my teenage self, my older sister was someone I really couldn’t stand. During my rebellious phase, whenever I had to fill out a form, I’d tell my mom, ‘I want to say we’re just two siblings’ (even though we were actually three), and she’d say, ‘That’s fine too,’ and let it slide.”

(Participant J)

Participant F, whose younger brother has had a physical disability since birth, watched her mother care for him from a young age.

“I wanted to help in some way, so I helped my mother with things like suctioning my brother and changing his diapers. But as I got into middle and high school, even though he was family, it started to feel a bit burdensome—I remember thinking, ‘Why is it always me?’”

At the same time, she felt that there was a difference in how she and her able-bodied brother perceived things.

“I don’t think my brother ever felt the need to hold back because he had a younger brother. He would casually invite friends over to our house. But whenever I invited someone over, I felt a strong sense of having to explain the situation and worried about what people might think”.

Some participants spoke openly with those around them about their siblings with disabilities, without hiding anything. Participant G had been aware of his younger sister’s developmental disability since childhood, but spending time with her felt natural, and he spoke about her to his friends without hesitation.

“My sister was happy when friends came over to stay, so I actually introduced them to her. I’ve never once felt the need to hide her. It doesn’t cause me any distress either”.

Although the participants may have felt confused about the changing perceptions of their siblings and their social standing, they had come to accept their circumstances and lived their lives accordingly.

#### 3.3.2. Subtheme 2: Deepening One’s Understanding of Disability and Welfare

“Deepening one’s understanding of disability and welfare” means that participants, through their work as helping professionals, have a desire to deepen their understanding of disability and welfare by re-examining unresolved issues, such as the sense of injustice they felt while living with a sibling with a disability.

Participant H shared an experience in which she felt frustrated because she was unable to do something to help her younger sister, who has autism and an intellectual disability, when she was being bullied. That experience influenced her career choice and motivated her to work with people with disabilities in their current position.

Participant E learned that her younger sister had an intellectual disability when she was in middle school. She spoke about the unease she had felt toward her sister up until that point and the guilt she felt for not having noticed her condition despite being so close to her. Carrying those feelings with her, she entered her current profession and now finds her work deeply rewarding.

“Although our roles may differ, I draw energy from the children and adults with disabilities I meet, and I’m happy to be able to talk with them. There are so many worlds I don’t know about, and I feel like it’s truly a continuous lifelong learning process. So, if you ask me if I’m satisfied, to be honest, I can’t say for sure. But in my current job, I want to continue to embrace the idea of lifelong learning”.

Some participants felt that their childhood experiences had not directly led them to pursue a deeper understanding of disabilities and social welfare. Participant C grew up in a household where her grandmother raised her and her two sisters equally, and she never felt uncomfortable spending time with her younger sister, who had an intellectual disability and physical impairments. After becoming a social worker, she began to consider how she could apply her personal experiences to her work.

Participant B, whose older brother has a progressive, incurable illness, learned of his diagnosis after starting her first job. After working in another helping profession, she decided she wanted to support people with disabilities and took her current position. She says she has deepened her understanding of disabilities and social welfare through her work with her clients, and even now, 20 years later, she still considers herself a student of this field. As for her older brother, who lives alone despite having a physical disability, she has simply been watching over him as things unfold.

#### 3.3.3. Subtheme 3: Discovery of One’s Identity

“Discovery of one’s identity” refers to the process in which, despite initially feeling uncertainty about one’s own identity while living with a sibling with a disability, one comes to clarify one’s own identity and the motivations for career choices through one’s role as a helping professional. From an early age, the participants had experienced feelings of alienation and conflict in their relationships with their sibling with a disability, both at home and at school. As they grew older, these feelings intensified through comparisons with others in group settings and through self-awareness.

Participant I felt shame and anger about living with her sister, who has cerebral palsy, and kept those feelings to herself, unable to talk about them with others. Believing that marriage would be a burden on her partner, she chose not to marry. She is currently involved in activities that support the families of people with disabilities.

“Because the number of people with disabilities is relatively small compared with the general population, I felt that we needed to take action and raise awareness, which is why I got involved in this work. I’ve decided to make this my life’s work”.

Participant H, whose older brother has a developmental disability, recalled watching as her brother was bullied at school. He struggled to maintain relationships, making it difficult for him to hold down a job. Although they lived together, she says she never really understood her brother; they would sometimes argue, and even now, in her twenties, she still feels a sense of distance between them. Despite this, she says she wants to help others who share her brother’s disability.

“Anyone can develop a mental illness, but I think it’s the kind of condition people don’t feel comfortable talking about with those around them. I suspect my brother feels isolated because of how society views him. I’m just guessing, but I think he must have wondered why he ended up with this illness. We weren’t close, but he’s still important to me. I want to work hard so that when the time comes for me to care for him in the future, he’ll be able to trust me. I’m glad I chose this career”.

By reflecting on their experiences with their siblings with disabilities—even though their family never forced them to do so—they came to recognize their own worth. Furthermore, by becoming a helping professional, they established their identity by discovering the strength to contribute to others and the significance of fulfilling a role within that context. The participants, especially those in the later stages of their careers, reflected on the sense of fulfillment they derive from their current jobs and linked it to their siblings.

“Of course, when I’m providing care and support, it’s not always smooth sailing. But I do find it rewarding to interact with the people I serve. I think the reason I feel that way is because my brother was there at the start—I suppose that’s why I decided to choose this path.”

(Participant A)

“I entered the social welfare field on a bit of a whim at first. It’s easy to just say it’s ‘rewarding,’ but in a way, I feel like I’ve truly found my calling here—there’s a strange sense of fate, a sense of wonder, and a desire to give it a try. So, looking back, I’m really glad I did.”

(Participant B)

In contrast, Participant C stated that she had never felt alienated or conflicted in her relationship with her disabled sister.

“My sister hasn’t been much help to my career. The support systems people use are completely different. I haven’t had any particular trouble accepting her disability. We’ve been together since we were little, so I’ve never really thought about it deeply, and I’ve never had to put up with anything. I think I only realized what’s truly important after I started working, and my sister’s presence has had a huge influence on that”.

### 3.4. Theme 3: Responsibility Toward Family

“Responsibility toward family” refers to the sense of duty that the participants developed from a young age after witnessing their families face difficult situations or feeling the expectation of their families. From childhood, the participants quietly observed how difficult it was for their parents to care for their sibling with a disability and to help them integrate into society, while harboring complex feelings about what to do. Participants were never pressured by the family to pursue a career in a human profession or to take care of their siblings. However, once they became a helping professional, their family came to rely on them and had high expectations. Through their work as helping professionals and their interactions with their clients’ families, they came to keenly realize the importance of supporting their own families. This theme comprises two subthemes: (1) facing family difficulties, and (2) expectations from the family.

#### 3.4.1. Subtheme 1: Facing Family Difficulties

‘Facing family difficulties’ meant that when difficult situations arose, the family was unable to cope on its own, and the participants could sense the fatigue and emptiness their family felt. In particular, when a sibling has a severe physical disability such as cerebral palsy, family members often provide round-the-clock care, and many of them carry a heavy burden and feel anxious. For the participants, this was a part of everyday life in their childhood. They tried not to burden their families with their own problems.

As Participant F’s younger brother, who has severe physical and mental disabilities, grew older, it became increasingly difficult for the family to live together, and they were forced to place him in a care facility. Under these circumstances, she tried to support the family while taking their situation into consideration. At the same time, however, she felt that she was unable to care for her brother herself.

“I don’t plan on taking care of my brother. I don’t want that to be my life, so I won’t be staying at home to look after him forever. Maybe it wasn’t so much that I wanted to take care of my younger brother as I wanted to help my mother. When it became clear that they could no longer care for my younger brother at home, my parents were torn for a long time. I told my mother that she had fulfilled her role, and that’s when I decided to take him to the hospital.”

(Participant F)

Even so, Participant F felt a strong desire to help her younger brother and to study with an eye toward the future. Although her parents told her not to worry about her brother when choosing a career and to pursue whatever path she wanted, she took her current job into consideration for the burden on her family.

#### 3.4.2. Subtheme 2: Expectations from the Family

‘Expectations from the family’ referred to the participants’ sense that their family members were relying on them to take care of their sibling with a disability in the future, as well as their desire to become a helping professional to satisfy those expectations. As discussed in Subtheme 2, “Not Forced,” the participants had not been directly asked by their families to take care of their siblings. However, some families hoped that after they passed away, their children would continue to look out for their siblings and never forget that they are family. The participants sensed this, and when reflecting on their career choices, they interpreted their decisions as an attempt to meet their parents’ expectations.

Participant D said she had been told that if her younger brothers were to enter a care facility, she would need to visit them from time to time and bring them necessary items, even if she got married and had a family of her own. Although she initially felt reluctant about this, she said she was prepared to do so.

“I guess there’s not much I can do about having to take care of my younger brother in the future, but since it’ll be helpful to him, I guess that’s okay”.

Participant H believed that caring for her brother with a disability was his calling.

“My family and my brother are part of my future. Including them in my plans is a big deal when I think about my future”.

Participant J majored in a different field in college, but following his mother’s advice to try to understand people with disabilities, he reconsidered his career path upon graduation and chose to become a nurse.

“I felt a bit ashamed because I had caused my mother trouble. Because I felt that shame, I thought that getting a job at a facility would be a way to make up for it”.

## 4. Discussion

The results of the present study revealed that, rather than choosing a career in a helping profession based on a clear and strong inclination or aspiration toward such work, participants tended to choose this career path as a result of being influenced by their family experiences and living circumstances from an early age. Previous research has pointed out that people with a sibling with a disability tend to worry about their sibling’s situation from an early age and are prone to feeling an excessive sense of responsibility (Bailey et al., 2020). It is also said that the siblings of people with intellectual or developmental disabilities play an important role within the family and often assume the responsibility of providing care as their siblings transition to life after high school. Furthermore, it has been reported that, in striving to maintain a balance among their siblings’ needs, family relationships, and their own well-being, they tend to suppress their own desires (Milo et al., 2021; Van Schoors et al., 2020). Among the participants in this study, many were living with their families when they made decisions about their education and choice of career, and the results indicated that they chose their path by adapting to the circumstances of their family and surroundings rather than actively pursuing their own aspirations. Adolescence and young adulthood are times when many young people experience conflicts regarding career choices and becoming independent; however, people with a sibling with a disability face the additional challenge of figuring out how to fulfill their roles and responsibilities toward their sibling (Nguyen et al., 2024). As a result of these accumulated experiences, the participants may have lived their lives suppressing their own needs both within and outside the home.

In this study, nine of the participants were women. This finding may reflect sociocultural expectations regarding caregiving roles. In Asian cultures in particular, the responsibility for caring for family members—including siblings with disabilities—is often viewed as a natural family obligation (H. Lee et al., 2025). Furthermore, the influence of traditional gender norms persists to some extent even today. Previous studies have reported that sisters are more likely than brothers to be involved in the care of siblings with autism and are more willing to provide care (Alon, 2022). It has also been shown that sisters tend to maintain stronger relationships with siblings who have disabilities (Prino et al., 2019). Given these findings, the high proportion of female participants in this study may reflect a situation in which women are more likely to internalize caregiving responsibilities within the family and to maintain ongoing relationships with their siblings with disabilities. It is conceivable that such experiences also influenced their orientation toward helping others, as well as the formation of their professional identity.

At the same time, when interpreting these results, it is necessary to take into account the differences related to disability. The siblings of the participants in this study had one or more intellectual, physical, or mental disabilities. Although it is difficult to compare them across the board, previous research suggests that siblings’ experiences vary depending on the type of disability. For example, Alon (2025) reported that siblings’ emotional experiences differ depending on whether their sibling has Down syndrome or autism, and that these experiences may also be influenced by the gender of the typically developing sibling. Therefore, both gender and the nature of the disability may influence how siblings perceive their roles within the family, their caregiving responsibilities, and their relationships with their siblings.

When participants recounted their early childhood experiences, they often expressed positive feelings towards their sibling with a disability. Previous research has shown that people with a sibling with a disability often experience negative emotions such as anger, jealousy, and frustration (Arcous et al., 2024; Yoo & Lee, 2023). However, it has been suggested that these emotions are often accompanied by guilt and may be suppressed by family dynamics and social expectations (Levante et al., 2025; Múries-Cantán et al., 2023). Furthermore, it has been reported that the expectation that those around them do not fully understand their situation and feelings, or will not understand them, can be a factor in refraining from speaking honestly, which can lead to the internalization of negative emotions (Hanvey et al., 2022). These findings suggest that the participants’ decision to pursue a career in the helping professions was driven by a desire for recognition from others—a need that had not been fully met during their childhood. It is believed that pursuing a career as a helping professional helped fulfill this need.

Similar to previous research (Stelter et al., 2026), the participants in the present study did not feel that their parents had placed direct expectations or pressure on them regarding career choices. However, given the context of their difficult family circumstances, they may have unconsciously internalized their parents’ expectations and the family’s needs. As a result, it is thought that some of the participants, despite their perception of “freely choosing,” may have actually had limited options, whereas others made choices within invisible constraints. Nevertheless, they have been engaged in helping professions for a long time and sought to deepen their understanding of disability and welfare by supporting clients and their families. It is possible that participants reinterpreted events they had initially experienced as constraints or inevitabilities through later reflection, situating them within the context of their own lives and career choices. This is also evident from the accounts of Participants A and B in Subtheme 3: Discovery of One’s Identity. This process may also explain the experiences of Participants I and J, who felt shame and anger and tried to hide the existence of their siblings from others. Although their childhood experiences were accompanied by emotional conflict, it can be said that, now in the later stages of their careers, these experiences have been reconstructed through introspection, leading to self-awareness and the discovery of their professional identities.

Considering this background, pursuing a career as a helping professional and acquiring specialized knowledge and perspectives on disability and social welfare likely provided participants with an opportunity to reinterpret the conflicts they had previously experienced and the emotional distance they felt from their siblings. By deepening their professional understanding, participants may have gained confidence in their interactions with siblings and family members, enabling them to build relationships more proactively. This process can be understood as a journey of self-actualization, enhancing their self-awareness as independent individuals and helping them reconstruct their sense of self-worth and life purpose. Furthermore, the experience of confronting the challenges faced by clients and their families as helping professionals and engaging in their care may have provided an opportunity to assign new meaning to past conflicts and suffering, serving as a catalyst for reinterpreting their own experiences.

### Strengths and Limitations

The discovery made in this study—that for siblings of people with disabilities, choosing a career in a helping profession is not merely a career choice, but rather a process of re-evaluating and reinterpreting past experiences, and reconstructing their own values and sense of purpose—highlights the novelty and significance of this study.

All participants in this study were Japanese. Therefore, there is a possibility that their cultural norms and values may be biased. Also, 9 out of 10 participants were women. This may reflect a sociocultural background in which women are more likely to take on responsibility and future caregiving roles (Alon, 2025; Kuo, 2014). Future research should employ sampling methods designed to increase male participation. Furthermore, longitudinal studies that examine changes in sibling relationships and the nature of challenges that arise over time may help in the development of lifelong support systems for siblings (Alon, 2026). Furthermore, while the siblings’ disabilities varied—ranging from intellectual and physical disabilities to mental disorders—it was difficult to clearly identify the impact of these differences. There is a need for more in-depth research focusing on siblings from a wider range of backgrounds, including diverse cultural backgrounds and various types of disabilities.

## 5. Conclusions

The results of this study revealed that participants did not choose a career in a helping profession based on a clear and strong inclination or aspiration, but rather as a result of being influenced by their family experiences and living circumstances from a young age. Participants spent their lives trying to maintain harmony with their families and harboring feelings that were difficult to articulate from a young age. Furthermore, it was suggested that pursuing a career in a helping profession is not merely a career choice but rather a process that prompts the re-evaluation and reinterpretation of past experiences, leading to the reconstruction of one’s own value and sense of purpose.

The findings of this study suggest the need to provide support for people who have a sibling with a disability. Specifically, possible approaches include establishing sibling support groups, providing opportunities for families to discuss future caregiving responsibilities, and implementing psychoeducational programs. Reflective supervision for helping professionals who have siblings with disabilities, as well as training modules within health, welfare, and education programs, would also be effective. Furthermore, incorporating these elements into continuing education could provide opportunities for helping professionals to reflect on and understand how the experience of having a sibling with a disability influences their values, professional identity, and approach to support. Moreover, this could help alleviate their psychological burden—which is often underrecognized—and promote self-understanding, potentially supporting the integration of personal experiences into their professional practice.

## Figures and Tables

**Table 1:** Interview guide.

・What is the most memorable episode from your time spent with a sibling with a disability? ・How did you feel you were perceived by those around you (e.g., family, friends, classmates) during your childhood? ・As someone with a sibling with a disability, what conflicts or joys did you experience regarding your own “role” and “way of being”? ・To what extent were you conscious of your sibling’s existence and experiences when considering your career path? ・When deciding to pursue a career in a “helping profession,” what were your family’s reactions, and what internal conflicts did you experience? ・How do your professional self and yourself as someone with a sibling with a disability influence your work? ・How has the “meaning” of your experiences as someone with a sibling with a disability changed throughout your career?

**Table 2:** Participant characteristics.

	Age (Years)	Gender	Occupation	Family Structure in Childhood	Sibling’s Age	Sibling’s Disabilities	Period of Living with Siblings	Care Level	Family Involvement
A	48	Female	Disability support coordinator	Father, mother, older brother, older brother (sibling), myself, and younger brother	40s (passed away 1 year ago)	Intellectual disability, physical disability	Until her older brother was 5 (after that, he entered a care facility)	Difficulty standing up, requiring full assistance	When they lived together, the family was responsible for helping the sibling with meals, bathing, and using the toilet. However, the sibling with disabilities became too big for home care to be feasible. The family visited the sibling at the care facility every week.
B	44	Female	Disability support coordinator	Father, mother, older brother (sibling), and myself	40s	Physical disability due to progressive disease	Until her older brother was 18 (after that, he lived alone with assistance)	Basically independent, but some movements require support	Her family kept their distance and watched over him while avoiding talking about her brother’s illness. As the disease progresses, they play a larger and larger role. She provides supervision and support.
C	26	Female	Medical social worker	Grandfather, grandmother, father, mother, myself, younger sister (sibling), younger sister	20s	Intellectual disability, physical disability	Until her younger sister was 12 (after that, she entered a care facility)	Moderate assistance is needed for movement or personal care	In childhood, her grandmother made her sibling’s meals and helped with bathing and toilet care. However, her sibling became too big for home care to be feasible. The family regularly visits the sibling at the care facility.
D	22	Female	Caregiver	Grandmother, father, mother, myself, and two younger brothers (twins, both with disabilities)	20s	Intellectual disability, physical disability	Until her younger brother was 18 (after that, he entered a care facility). Second younger brother is still living at home.	Moderate assistance is needed for movement and personal care for the younger twin; the older twin is able to manage daily life almost independently	During her childhood, she helped her mother and, as the eldest sister, took responsibility for cooking meals for her younger brothers and assisting them with bathing and toileting. The older twin is currently hospitalized, and the younger twin is worried.
E	27	Female	Nurse	Father, myself, younger sister (sibling)	20s	Intellectual disability	Until E was 18 (her younger sister is still living at home)	Nearly bedridden and in need of total assistance	As a child, she did not get along well with her sibling, and her family was not very involved. After the illness was confirmed, the family began to participate in monitoring and watching.
F	23	Female	Nurse	Father, mother, older brother, myself, younger brother (sibling)	20s	Severe physical and intellectual disabilities	Until her younger brother was 18 (after that, he was hospitalized)	Nearly bedridden and in need of total assistance	During her childhood, her mother mainly took the lead in caring for her younger brother. As the older sister, she helped their mother and took care of her younger brother. Her involvement decreased during adolescence. Currently, due to the worsening of her younger brother’s condition, he is hospitalized.
G	27	Female	Social worker in a long-term care facility	Father, mother, myself, younger sister (sibling)	20s	Autism, intellectual disability	Living together	Unable to stand independently; requires full assistance	Ever since childhood, her family has supported her younger sister in all aspects of daily life, including meals, cleaning, and personal hygiene. Even now, she continues to care for her younger sister as a family member. She has no hesitation in caring for her sister and enjoys spending time with her.
H	26	Female	Psychiatric social worker	Grandfather (who died when H was in elementary school), grandmother (who died 2 years ago), father, mother, older brother (sibling), myself	30s	Developmental disorder, mental illness	Until H was 18 (her older brother is still living at home)	Able to manage daily life almost independently	As a child, she did not get along with her brother, who also had a strained relationship with the rest of the family. After his illness was diagnosed, the family became involved in monitoring and caring for him.
I	61	Female	Social labor consultant assistant	Grandfather, grandmother, father, mother, older sister (sibling), myself, younger brother	70s (passed away 1 year ago)	Cerebral palsy, severe physical and intellectual disabilities	Until her older sister was 12 (after that, she entered a care facility)	Nearly bedridden and in need of total assistance	As children, her parents prepared meals and helped with bathing and toilet care. The siblings helped out to the extent they could. However, her sibling became too big for home care to be feasible. After her sibling was institutionalized, her parents frequently went to the care facility for interviews. As the parents aged, she and her younger brother visited their sibling in the care facility and took on the role of watching over her.
J	56	Male	Nurse	Father, mother, older sister (sibling), sister, myself	60s	Intellectual disability, physical disability due to post-encephalitis sequelae	Until his older sister was 15 (after that, she entered a care facility)	Nearly bedridden and in need of total assistance	During his childhood, his mother prepared meals and helped with bathing and toilet care. Due to the age gap between him and his older sisters, he was not directly involved in caring for his sibling. At school, he hid the existence of his sibling from his classmates. When his sibling had grown too big for home care to be feasible, she was institutionalized. Her mother frequently visited his sibling at the care facility. Currently, he works at the same facility and watches over others with the same condition.

**Table 3:** Example coding tree.

Participants’ Description	Code	Subtheme	Theme
I never really thought about pursuing my current career when I was growing up. I listed it as my third choice on the career preference survey in high school… I just sort of thought, “Maybe I’ll go into social welfare…” and that’s just how it turned out.	Somehow familiar and close	Not having strong motivation	By chance
I don’t think my parents had much in the way of expectations regarding my future. My mother, in particular, always told my siblings and me to follow our own paths.	Not that my family told me to, but I decided to	Not forced	
To be honest, when I was little, I used to think that Grandma should go to the hospital (to take care of my little brother) and Mom should stay home.	Feeling angry or embarrassed	Conflicts from an early age	Solving personal challenges
In my current job, I’ve recently started to question the employment situation for people with disabilities—specifically their low wages and limited options. I’ve been thinking about what can be done to address this.	Thinking about work-related matters	Deepening one’s understanding of disability and welfare	
Because the number of people with disabilities is relatively small compared with the general population, I felt that we needed to take action and raise awareness through our family’s efforts, which is why I got involved in this work. I’ve decided to make this my life’s work.	Feeling a sense of fulfillment in work	Discovery of one’s identity	
My younger brother has been bedridden and unable to speak since infancy. At home, my mother took the lead in caring for him. We all took turns going to the hospital… and my mother was often gone for a long time…	Understanding the severity of the disability and the challenges faced by the family	Facing family difficulties	Responsibility toward family
My parents have told me about the future. They say that, eventually, my younger brother will have to go into a facility or something, or else he’ll become a burden, and my older brother might still be at the hospital. They’ve told me that I should visit them from time to time and bring them anything they might need, even if I get married and have a family of my own. I guess there’s not much I can do about having to take care of my younger brother in the future, but since it’ll be helpful to him, I guess that’s okay.	Wanting to live up to my family’s expectations	Expectations from the family	

## Data Availability

The data presented in this study are available on request from the corresponding author due to privacy or ethical restrictions.
